# Rationale, design and methods of VA-BRAVE: a randomized comparative effectiveness trial of two formulations of buprenorphine for treatment of opioid use disorder in veterans

**DOI:** 10.1186/s13722-022-00286-6

**Published:** 2022-01-31

**Authors:** Ismene Petrakis, Sandra A. Springer, Cynthia Davis, Elizabeth Ralevski, Lucy Gu, Robert Lew, John Hermos, Melynn Nuite, Adam J. Gordon, Thomas R. Kosten, Edward V. Nunes, Robert Rosenheck, Andrew J. Saxon, Robert Swift, Alexa Goldberg, Robert Ringer, Ryan Ferguson

**Affiliations:** 1 Department of Psychiatry, Veterans Affairs Connecticut Healthcare System, West Haven, CT USA; 2 Department of Psychiatry, Yale School of Medicine, New Haven, CT USA; 3 Section of Infectious Disease, Department of Internal Medicine, Veterans Affairs Connecticut Healthcare System, West Haven, CT USA; 4 Section of Infectious Disease, Department of Internal Medicine, Yale School of Medicine, New Haven, CT USA; 5 US Department of Veteran Affairs, Cooperative Studies Program Coordinating Center (CSPCC), Boston, MA USA; 6 Department of Psychiatry, Harvard Medical School, Boston, MA USA; 7 Center for Healthcare Organization and Implementation Research, VA Boston Healthcare System, Jamaica Plain, Boston, MA USA; 8 Department of Public Health, Boston University, Boston, MA USA; 9 Section of General Internal Medicine, Department of Medicine, School of Medicine, Boston University, Boston, MA USA; 10 Department of Internal Medicine, VA Boston Healthcare System, Jamaica Plain, Boston, MA USA; 11 Informatics, Decision-Enhancement, and Analytic Sciences (IDEAS) Center, VA Salt Lake City Health Care System, Salt Lake City, UT USA; 12 Division of Epidemiology, Department of Internal Medicine, Program for Addiction Research, Clinical Care, Knowledge and Advocacy (PARCKA), University of Utah School of Medicine, Salt Lake City, UT USA; 13 Department of Psychiatry, Michael E. DeBakey Veterans Affairs Medical Center, Houston, TX USA; 14 Department of Psychiatry, Baylor College of Medicine, Houston, TX USA; 15 Department of Psychiatry, Columbia University Medical Center, New York, NY USA; 16 Center of Excellence in Substance Addiction Treatment and Education, VA Puget Sound Health Care System, Seattle, WA USA; 17 Department of Psychiatry and Behavioral Sciences, University of Washington School of Medicine, Seattle, WA USA; 18 Providence Veterans Affairs Medical Center, Providence, RI USA; 19 Department of Psychiatry and Human Behavior, Center for Alcohol and Addiction Studies, Brown University Warren Alpert Medical School, Providence, RI USA; 20 US Department of Veteran Affairs, Cooperative Studies Program Clinical Research Pharmacy Coordinating Center, Albuquerque, NM USA

**Keywords:** Opioid use disorder, Buprenorphine, Comparative effectiveness trial, Veterans, Injectable

## Abstract

**Background:**

To address the US opioid epidemic, there is an urgent clinical need to provide persons with opioid use disorder (OUD) with effective medication treatments for OUD (MOUD). Formulations of sublingual buprenorphine/naloxone (SL-BUP/NLX) are considered the standard of care for OUD including within the Veterans Healthcare Administration (VHA). However, poor retention on MOUD undermines its effectiveness. Long-acting injectable monthly buprenorphine (INJ-BUP) (e.g., Sublocade®) has the potential to improve retention and therefore reduce opioid use and overdose. Designing and conducting studies for OUD pose unique challenges. The strategies and solutions to some of these considerations in designing Cooperative Studies Program (CSP) 2014, Buprenorphine for Treating Opioid Use Disorder in Veterans (VA**-**BRAVE), a randomized, 20-site, clinical effectiveness trial comparing INJ-BUP to SL-BUP/NLX conducted within the VHA may provide valuable guidance for others confronted with similar investigation challenges.

**Methods:**

This 52-week, parallel group, open-label, randomized controlled trial (RCT) evaluates the comparative effectiveness of two current FDA-approved formulations of buprenorphine: (1) daily SL-BUP/NLX vs. (2) monthly (28-day) INJ-BUP for Veterans with moderate to severe OUD (n = 952)**.** The primary outcomes are (1) retention in MOUD and (2) opioid abstinence. Secondary outcomes include measures of other drug use, psychiatric symptoms, medical outcomes including prevalence rates of HIV, hepatitis B and C as well as social outcomes (housing instability, criminal justice involvement), service utilization and cost-effectiveness. Special considerations in conducting a comparative effectiveness trial with this population and during COVID-19 pandemic were also included.

**Discussion:**

The evaluation of the extended-release formulation of buprenorphine compared to the standard sublingual formulation in real-world VHA settings is of paramount importance in addressing the opioid epidemic. The extent to which this new treatment facilitates retention, decreases opioid use, and prevents severe sequelae of OUD has not been studied in any long-term trial to date. Positive findings in this trial could lead to widespread adoption of MOUD, and, if proven superior INJ-BUP, by clinicians throughout the VHA and beyond. This treatment has the potential to reduce opioid use among Veterans, improve medical, psychological, and social outcomes, and save lives at justifiable cost.

*Trial registration* Registered at Clinicaltrials.gov NCT04375033

## Background

There are many challenges and considerations to designing and conducting comparative effectiveness studies for opioid use disorder (OUD). The strategies and solutions to some of these considerations in the design of a multi-site, national comparative effectiveness trial may provide valuable guidance for others confronted with similar challenges. VA Cooperative Studies Program #2014 (CSP #2014) is the first long-term, direct effectiveness comparison trial of monthly injectable formulation of buprenorphine (INJ-BUP) to daily formulation of buprenorphine + naloxone (SL-BUP/NLX) and the first to be conducted in the Veterans Healthcare Administration (VHA) system among Veterans with OUD.

In October 2017, the US declared that the rise in heroin and fentanyl use [1, 2], and dramatic increase of opioid overdose deaths [3], including among Veterans [4], was a national epidemic and public health emergency [5, 6]. From October 2018–2019, over 47,000 individuals died of opioid overdoses in the United States [7, 8]. Early data emerging in 2020 suggest that overdose deaths continue to climb during the COVID-19 pandemic [9].

The number of individuals diagnosed with OUD also has increased with the rise in opioid misuse. It is estimated there were 2.1 million Americans diagnosed with OUD in 2018 [10]. The number of Veterans diagnosed with OUD who are seeking treatment from the VHA has also dramatically increased in the past few years, similar to that seen in the general community. There was a 131% increase in OUD diagnoses from 2001 (27,840 cases of OUD) to 2015 (64,373 cases), with 69,142 Veterans diagnosed with OUD in fiscal year 2017 and now over 80,000 diagnosed with OUD [5, 11]. As in the general population, OUD among Veterans is associated with housing instability, mental health diagnoses, suicide, and criminal justice involvement [12].

The most effective treatment for OUD are medications of three types: the full opioid agonist methadone, the partial agonist buprenorphine, and the antagonist naltrexone in extended-release injectable formulation (XR-NTX) [13–16]. These medications have been shown to reduce relapse to opioid use, overdose, HIV and HCV transmission, and improve other health outcomes [17, 18]. Overall, clinic-based daily SL-BUP/NLX treatment has become the treatment of choice for OUD because it has a favorable safety profile, can be administered in community settings that include primary care provider offices and does not require detoxification prior to initiation [14]. Clinic-based treatment with SL-BUP/NLX is the standard of care for OUD within the VHA [11].

Despite evidence that daily SL-BUP/NLX is effective, it is discontinued at high rates, undermining its effectiveness. Several reviews show that buprenorphine retention at 6 months is estimated at less than 40% [19, 20]*.* A 2020 systematic review reported retention rates falling from 58% at 6 months to 38.4% at 3 years [21, 22] .In an earlier study with a Veteran population, retention rates fell from 61.6% at 1 year to 31.83% at 3 years [22]. More recently, data from the VA Center of Excellence in Substance Use Disorder Treatment and Education (unpublished, 2017) shows only 35% of Veterans were retained in SL-BUP/NLX treatment at 12 months after initiation; with the highest dropout rate (almost 25%) within the first 30 days.

Long-term opioid abstinence is generally achieved through long term treatment with medications for opioid use disorder (MOUD), including SL-BUP/NLX [23, 24], and there is high risk for relapse and overdose particularly during the first 30 days after discontinuation [25]. Maximizing retention is thus of great clinical importance, as good retention is associated with lower mortality rates, lower emergency room utilization [26], and improvement in domains including risk for and treatment of HIV [27, 28].

Injectable buprenorphine (e.g., Sublocade®) is a monthly alternative to the daily form of SL-BUP/NLX for treatment of persons with moderate to severe OUD. The monthly formulation provides a steady plasma concentration and thus may be more favorable for persons who have difficulty adhering to daily SL-BUP/NLX. Sublocade® has already been shown to be well tolerated and both available doses are associated with a higher percentage of abstinence (41.3%; 42.7%) compared to placebo (5.0%) [29]. Data presented by Indivior pharmaceutical company, the manufacturer of Sublocade®, show it was associated with retention in treatment at 60% at 6 months and 12 months [29] and in their final published trial data in Lancet 2019 [29] they report an overall retention rate as 60% in the 24 week trial. While it has not yet been directly compared to SL-BUP/NLX, a retrospective chart review comparing retention between the 2 formulations showed a significantly higher number of visits for those on Sublocade® vs. sublingual buprenorphine, but no significant difference in retention in 6 months [30].

**Fig. 1:**
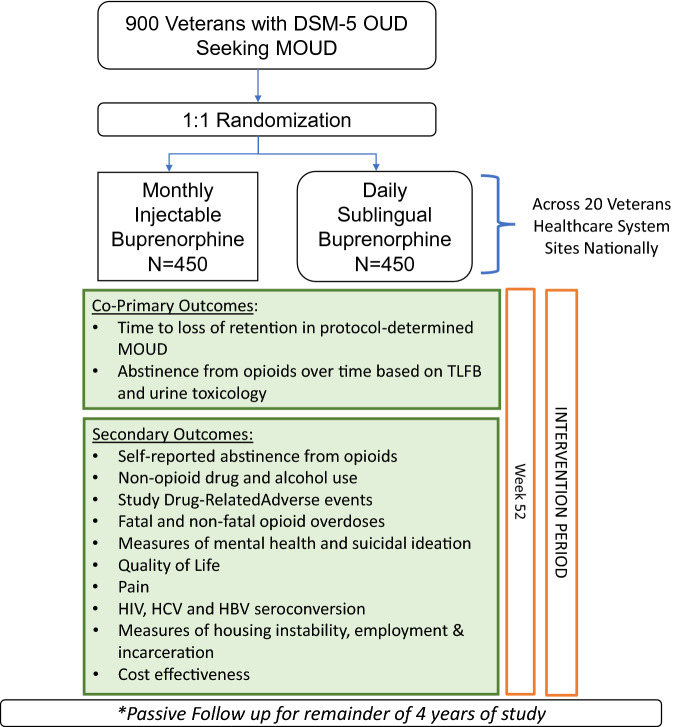
CSP2014: VA-BRAVE study design. *OUD* opioid use disorder, *MOUD* medication treatment for OUD, *MOUD* medication for opioid use disorder, *TLFB* timeline follow-back

Therefore, we designed CSP #2014 “Buprenorphine for Treating Opioid Use Disorder in Veterans (VA-BRAVE)” (Fig. 1), a 52-week randomized controlled study to compare the effectiveness of the long acting monthly injectable formulation of buprenorphine versus the standard of care sublingual formulation, administered as part of routine outpatient care to 952 Veterans across 20 sites in the VHA. There are 2 co-primary outcomes and the study was designed to determine if long-acting injectable buprenorphine is superior to the sublingual form in retention on medication and abstinence from opioids. In addition to the study design and methods proposed to conduct this clinical trial, this manuscript describes the practical, safety and ethical considerations of working with participants who have OUD, selection of the outcomes, and duration of the trial among other issues. We discuss considerations for special sub-populations and events within the study population including pregnancy, incarceration, new onset infectious disease and overdose. Lastly, before the trial was initiated, a worldwide pandemic with COVID-19 effectively interrupted clinical care nationally; considerations on how to proceed while protecting participant and staff safety were developed and are reviewed.

## Methods

### Study population

The eligibility criteria to participate in this trial (Table 1) are deliberately broad to maximize the inclusion of Veterans in order to assess the co-primary outcomes (retention in MOUD and opioid abstinence) and to mirror ‘real world’ patients with OUD who present for MOUD in VHA clinical settings. Participants who have moderate-to-severe OUD [Diagnostic Statistical manual-5th Edition (DSM-5)] [31] who are entering a new episode of care and who are 18 years or older are eligible. This includes participants with psychiatric, substance use and medical comorbidities that are typical in the VHA OUD population. Participants previously maintained on buprenorphine or other forms of MOUD are permitted to participate, but in order to be eligible for this trial they must be initiating buprenorphine as a *new episode of care* at the time of study enrollment (< 30 consecutive days on MOUD treatment). As a real-world effectiveness trial, there are no restrictions in participation in psychosocial programs and other formal supports. Service utilization will be assessed as a secondary objective.

**Table 1:** CSP#2014: VA-BRAVE eligibility criteria

Inclusion criteria	Exclusion criteria
Use of opioids within 30 days prior to consent *or* within 30 days prior to entry into a supervised setting Meets DSM-5 criteria for moderate to severe OUD Referred to/seeking treatment for OUD New episode of care, defined as requiring induction on buprenorphine OR taking a form of prescribed medication treatment for OUD continuously for < 30 days prior to consent	Veterans < 18 years of age Females unwilling to practice an effective method of birth control for the duration of the study History of significant adverse effects from buprenorphine and/or naloxone Recent suicidal or homicidal ideation or psychosis that requires hospitalization Unable or unwilling to provide consent Meets criteria for current DSM-5 sedative hypnotic use disorder Pending felony charges Conditions which, in the judgement of the Local Site Investigator, make it unlikely the patient can participate in or complete the 52-week active phase of the study, including *current* moderate-to-severe COVID-19 symptoms with a risk of intubation or critical illness Is *actively* participating in another interventional clinical trial for which a waiver of dual-enrollment with VA-BRAVE has not been obtained

The exclusion criteria are also minimal to enhance generalizability, while maximizing patient safety. These include conditions (e.g., psychiatric conditions) requiring a higher level of care or medical conditions that preclude the use of buprenorphine. While participants are expected to have comorbid substance use disorders, whether to exclude participants using sedative hypnotics was carefully considered. Recent guidance from the American Society of Addiction Medicine (ASAM) [32] encourages treatment with buprenorphine even in those using sedative hypnotics since buprenorphine protects against risk of overdose from opioids. In this study, those participants at highest risk of combining medications, defined as those with a DSM-5 diagnosis of current sedative hypnotic use disorder are excluded.

Similarly, it is expected that participants will have medical comorbidities including hepatitis B and C and HIV. Only those participants for whom buprenorphine is medically contraindicated or who require intensive medical management (e.g., Childs-Pugh Class C cirrhosis) are excluded. Those with pending felony charges are also excluded due to likelihood of incarceration interfering with participation. In addition, in response to the COVID-19 pandemic, exclusion criteria exclude those with current moderate to severe COVID-19 symptoms who were at risk of intubation or critical illness. Those participants are able to join if they meet eligibility criteria after they recover from the acute phase of the infection.

**Fig. 2:**
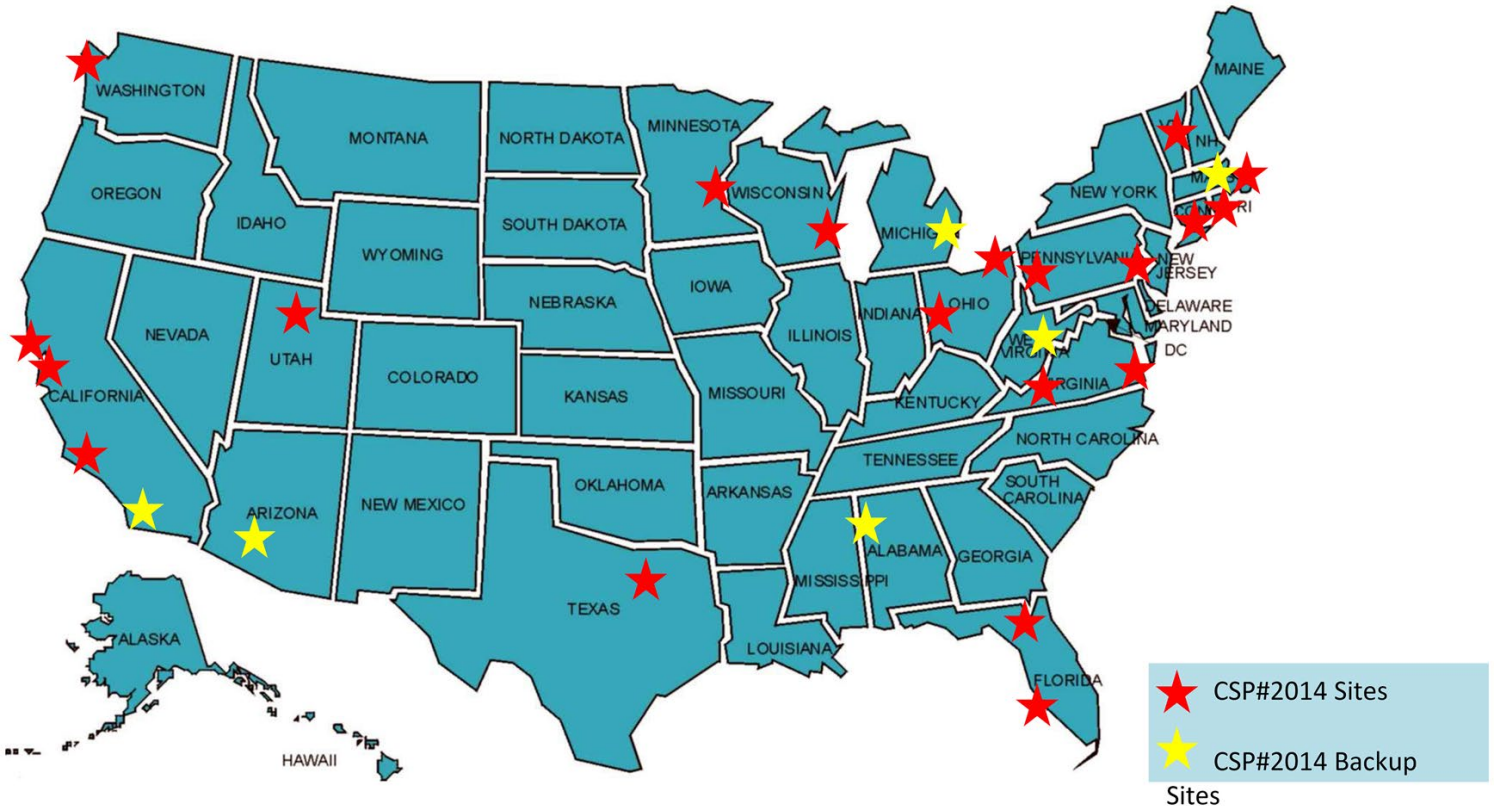
CSP2014: VA-BRAVE map of study sites. Original active sites: Bay Pines, FL; Boston, MA; Cleveland, OH; Dallas, TX; Dayton, OH; Gainesville, FL; Hampton, VA; Long Beach, CA; Milwaukee, WI; Palo Alto, CA; Philadelphia, PA; Pittsburgh, PA; Providence, RI; Salem, VA; Salt Lake City, UT; San Francisco, CA; West Haven, CT; Seattle, WA; White River Junction, VT. Back-up sites: Bedford, MA; Detroit, MI; Huntington, WV; Phoenix, AZ; San Diego, CA; Tuscaloosa, AL

Twenty geographically diverse sites were chosen across the United States with consideration for sites with facilities capable of supporting CSP research as well as those areas hard-hit by the opioid epidemic as shown in Fig. 2.

### Participant identification and consent

This study is approved by the VA-Central Institutional Review Board (CIRB). Early identification, screening, and contact of patients seen by the medical center’s MOUD clinic and substance use disorder specialty clinics, mental health clinics, primary care and sub-specialty medical clinics and inpatient units responsible for the evaluation and treatment of patients with likely or confirmed diagnoses of OUD are key to the study’s success. CIRB-approved flyers and information sheets were developed to reach a broad and inclusive audience at local sites and result in patient-initiated contact with the local site study team.

Written consent for further screening and study participation is obtained in-person by an authorized member of the study team. Patients ineligible or unwilling to participate at any point in the recruitment process or who withdraw consent are referred for appropriate treatment via a warm handoff based upon consultation with the site study clinician.

### Devising clinically relevant treatment arms

The goal of CSP 2014, VA-BRAVE, is to evaluate whether the long acting injectable buprenorphine is more effective than the current standard of care, sublingual buprenorphine, and whether its cost effectiveness merits its adoption. One design challenge was to isolate the route of administration as the independent variable and, in order to satisfy equipoise, careful consideration was taken in terms of induction processes, dose, and frequency of medication prescription.

Participants who meet eligibility criteria are initiated on SL-BUP/NLX as soon as clinically possible in accordance with good clinical practice. Buprenorphine is prescribed during an induction phase with SL-BUP/NLX starting at a dose of 2 mg and then increased as needed for stabilization of opioid withdrawal symptoms. This induction procedure is consistent with clinical care as described in clinical practice guidelines [33] including SAMHSA TIP 63 practice guidelines [34]. The target maintenance dose is 16 mg to 24 mg titrated by day 3, with room for clinical flexibility defined as not more than 30 days from induction. This study allows for rapid initiation of INJ-BUP in the service of getting patients stabilized on treatment as soon as possible. Potential participants who are already taking a form of buprenorphine for less than 30 days or who are in the process of being clinically inducted are also eligible for the study and bypass protocolized induction procedures. As soon as a maintenance dose is identified, the participant is ready for randomization.

Once reaching the target dose, eligible participants are randomized 1:1 to receive either continued daily SL-BUP/NLX at the dose identified in the induction period or to receive monthly subcutaneous abdominal INJ-BUP with a target dose of 300 mg, with the option to use 100 mg dose. Randomization is performed centrally by the CSP Clinical Research Pharmacy Coordinating Center (CSPCC) using an interactive web-based randomization program that allows for randomization of participants in real time by authorized study team members at each site who submit a randomization request form. The study participant is allocated to the appropriate treatment arm according to the randomization schema and a certificate is generated. The certificate is used by the local site investigator and research pharmacist to direct study drug dispensing. Participants are informed of their randomized treatment assignment during their visit, and prior to initiation of either SL-BUP/NLX or INJ-BUP.

This is an open label comparative effectiveness study so neither staff nor participants are blinded to treatment assignment.

### Study dose

**Fig. 3:**
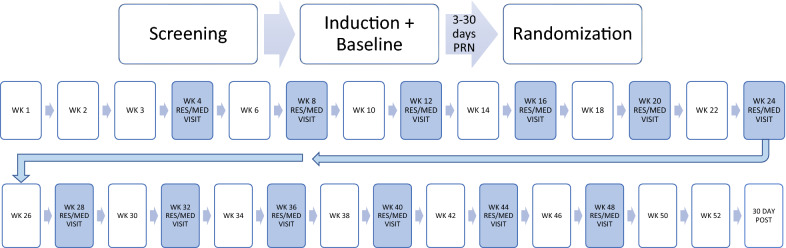
CSP2014: VA-BRAVE timeline. PRN: as needed; WK: week; RES/Med VISIT: Research/Medication Administration Visit; 30 Day POST: 30 day post study safety monitoring period. *At each visit, research assessments are collected. For more information on the schedule of assessments, refer to Table 1

Study drug is prescribed for a treatment course totaling 52 weeks in either 28-day prescriptions of daily SL-BUP/NLX (target dose 16–24 mg) or 28-day INJ-BUP (target dose 300 mg) injections, a formulation which provides steady blood level over 28–30 days. While two doses of INJ-BUP are available, 100 mg and 300 mg, the 300 mg dose was chosen as a target dose as it delivers a far more adequate steady state mean blood level and has been associated with better opioid abstinence outcomes than the 100 mg dose for those who inject heroin [35]. The SL-BUP/NLX formulation used in this study is a sublingual film formulation across all sites. The dose range of 16–24 mg is the standard dose recommended for clinical practice. The prescribing physician can make adjustments in dose for the SL-BUP/NLX following standard clinical practice. For those randomized to INJ-BUP, the 300 mg dose is the target dose; however, the prescribing physician can lower the dose to 100 mg after the first injection depending on patient preference or if clinically indicated (e.g., opioid agonist side effects). In order to satisfy equipoise, participants randomized to the SL-BUP/NLX arm receive a 28-day take-home supply; while those in the INJ-BUP arm receive monthly INJ-BUP administered in the clinic at 28-day intervals. The study visits as depicted in Fig. 3 are identical between groups, and all participants regardless of their assigned treatment arm, receive a Medication Management (MM) session at each 28-day study visit (described below). Each site has a local site investigator and a study team; all study-related drugs are prescribed and administered by a clinician(s) (e.g., MD, NP, DO, PA, RN). Site investigators are mostly psychiatrists but also include primary care physicians; all are clinicians working in substance abuse clinics or in settings in which they are treating patients with opioid use disorder. Site investigators were chosen based on their experience with the patient population and with research (or with the availability of local research mentorship).

### Study length

VA-BRAVE is a 52-week study that includes scheduled receipt of study medication and active follow-up. Many studies of OUD are limited to 12 or 24 weeks of active treatment; given that OUD can be chronic and relapsing, longer studies are needed to understand the clinical benefit of buprenorphine over time. This 52-week study will be better able to evaluate retention on study drug and opioid abstinence. In addition, there is an additional passive follow-up period, using the electronic medical record of up to 10 years (from date of first participant randomized), to assess longer-term patterns of service use including medications and hospitalizations.

### Retention

Local staff will maintain participant engagement via periodic phone calls to remind them about visits, for check-ins between visits, to follow-up on missed visits, and to obtain relevant study data if necessary. Participant-provided contact forms facilitate this outreach. Participants will be notified that those listed may be contacted but information regarding study participation will not be disclosed. Local staff will update this form as needed throughout the study. Participants will not be considered “lost to follow-up” until their week 52 visit, so there is always the opportunity to re-engage with the study after a lapse.

### Ethical design and care

The study design has particular strengths that contribute to its scientific value and ensure ethical care. The choice of primary and secondary outcomes provides broad, patient-centered results, critical to the lives of Veterans seeking medication treatment for OUD. The study’s exclusion criteria are minimal in order to make the results generalizable to a wide spectrum of Veterans seeking medication treatment for OUD (Table 1). Additionally, the induction phase preceding randomization follows established guidelines to engage participants in MOUD as early in treatment as possible.

Participants with OUD are at risk for opioid overdose. To mitigate this risk, study procedures ensure all participants receive Overdose Education and Naloxone Distribution (OEND) consistent with usual good clinical care practices for Veterans with OUD. In addition, all participants are provided Medication Management, a 15-min counseling session that recommends opioid and other substance use abstinence and adherence to MOUD.

### Outcomes

One strength of this study is its use of two co-primary outcomes, retention and opioid abstinence, chosen because they are clinically meaningful, practical, patient-centered, and appropriate for a 52-week, large-scale comparative effectiveness trial.

*Retention on treatment* Retention on MOUD is a highly sensitive indicator of effective treatment, as discontinuation is strongly associated with recurrence to use of opioids, risk for overdose, transmission of bloodborne infectious diseases, and incarceration. Retention is defined as time from randomization to the first period of missed study-prescribed drug coverage lasting at least 4 weeks. This outcome is accurately measurable, unaffected by loss to follow-up, and highly indicative of clinical benefit.

*Opioid abstinence* reflects direct opioid use and will be indicated by self-report opioid abstinence using the systematic timeline follow-back (TLFB) [36] method *and* urine toxicology (UTOX) negative for opioids across 28 timepoints. The National Institute on Drug Abuse (NIDA)-funded Clinical Trials Network investigators have recommended using the method of self-report plus toxicology as a standard in substance use disorder trials.

Self-reported drug use is collected using the TLFB calendar method, in which study personnel record the participants’ reported opioid use for each day since the last study visit. The TLFB is reliable and valid when used by trained interviewers and when there is no penalty for reporting use of drugs. The advantage to this method is that data are collected retrospectively to the last visit, so a missed appointment does not necessarily result in missing self-report data; limitations include reliance on self-report and lack of objective measurement. For that reason, a biological measure, with UTOX as standard, is also collected as an indicator of opioid abstinence. UTOX for this study is a UTOX-13 panel from a centralized lab, Redwood Toxicology, and collected at each study visit as indicated in Fig. 3 and the Schedule of Assessments in Table 2 and measures opioid drug use including oxycodone/noroxycodone, benzodiazepines, ethyl glucuronide, methadone, fentanyl, buprenorphine, tramadol, methylene-dioxy-methamphetamine (MDMA), amphetamines, cocaine metabolite, opiates, cannabinoids, and THC/creatinine ratio. UTOX screening is a routine part of buprenorphine maintenance treatment. UTOX screening methods have the issue of how to handle missing data [37]. For this trial, the conservative method that assumes missing UTOX is indicative of use is used (although participants may miss appointments for other reasons) and is consistent with most published research. Both the TLFB and UTOX data must be negative for opioid use to deem a participant abstinent.

**Table 2:** CSP#2014, VA–BRAVE study assessments

Assessment	Screening	Baseline	Randomization	Weeks 1–3	Medication-research visits	Non-medication visits	End of study visit (week 52)
Biological assessments							
Urine pregnancy test	√	√	√		√		√
Physical exam	√				Week 24		√
Liver and kidney function, complete blood count (CBC)	√				Weeks 4, 12, 24		√
Electrocardiogram (EKG)	√				Week 24*		
Urine toxicology test		√		√	√	√	√
Blood HIV, hepatitis B (HBV), and hepatitis C (HCV)		√			Week 24		√
Blood buprenorphine and norbuprenorphine		√			Week 24		√
Interviewer administered assessments							
The MINI International Neuropsychic Interview (MINI) [39]	√	√					√
Clinical Opiate Withdrawal Scale (COWS) [40]		*	√	*	*	*	*
Clinical Institute Withdrawal Scale (CIWA-Ar) [41]	√	*	*	*	*	*	*
Patient Health Questionnaire-9 (PHQ-9, item 9 only) [42]	√			√	√	√	√
Timeline follow-back [43]		√		√	√	√	√
Concomitant medications		√		√	√		√
Opioid craving and overdose form		√		√	√		√
PEG3 (pain, enjoyment, general activity)		√		√	√		√
Service utilization review form (SURF)		√			√		√
HIV risk behaviors		√			Weeks 12, 24, 36		√
Criminal justice involvement		√			Week 24		√
Alcohol Use Disorders Identification Test (AUDIT) [44]		√					√
Demographics and military history		√					
Modified substance use and medical history		√					
Columbia Suicide Severity Rating Scale (C-SSRS) [45]		√					
Study Medication Adherence Visual Analog Scale (VAS)				√	√		√
Serious/adverse event		*	*	*	*	*	*
Self-report assessments							
Veterans Rand-12		√			√		√
Patient Health Questinnaire-9 (PHQ 9)		√			Weeks 12, 24, 36		√
Posttraumatic Stress Disorder Checklist for DSM-5 (PCL-5)		√			Weeks 12, 24, 36		√
COVID-19 Questionnaire		√			Weeks 12, 24, 36		√

√ = collected at each timepoint; * = collected only when necessary; please note some assessments are collected only during weeks indicated in the table

### Secondary objectives

Secondary objectives include determining whether the use of INJ-BUP is associated with better outcomes than SL-BUP/NLX in: opioid craving; use of other illicit substances and alcohol; preventing opioid overdose; reducing mental health symptoms (including depression, PTSD, and suicidality; homelessness); incarceration and criminal legal involvement; self-reported risky sexual and injection drug use behaviors and the incidence of new cases of HIV, HBV, and HCV. In addition, the total cost impact of INJ-BUP and cost-effectiveness from the perspective of VHA and of society will be evaluated. We will thus also assess non-VA service utilization, especially since the MISSION ACT [38], allows a Veteran to receive care from a community provider paid for by the VA. Table 2 summarizes the Schedule of Assessments including self-report and biological measures and their administration times. Data will also be captured to identify reasons for missed study injections as well as overall study visits.

## Special circumstances

The following circumstances are anticipated for this population and considerations were made regarding study participation, procedures, and follow-up related to each.

### Incarceration

Participants cannot be interviewed, have study procedures administered or receive study drug while incarcerated. However, if an incarceration episode has been concluded when discovered, and no immediate future risk of incarceration is present, then continuation of participation is reasonable. The study team may review the medical records of participants who are incarcerated. Participants with ‘brief’ incarceration periods, defined as a period of less than the remainder of the participant’s 52-week study period, may be eligible to continue study participation if they so choose after they are released back to the community.

### Non-fatal and fatal accidental overdose

Information regarding accidental drug poisoning (i.e., overdose) will be obtained and tracked via participant self-report, naloxone distribution records, hospital records both within and outside VHA, as well as CDC accidental drug poisoning data, if possible. If a participant experiences non-fatal accidental drug poisoning, this would not necessarily impact eligibility.

### Newly diagnosed HIV/HBV/HCV infections

Information regarding HIV/HBV/HCV will be obtained and tracked via participant self-report, HIV/HBV/HCV blood tests at periodic study visits (see Table 2), and hospital records both within and outside VHA. If a participant has a newly diagnosed HIV/HBV/HCV infection, this would not necessarily impact eligibility or continued study participation.

### Pregnancy

Urine pregnancy testing will be conducted on all Veterans of childbearing potential at screening, induction, baseline, and randomization as well as all follow-up visits during which study medication is prescribed/administered as denoted in Table 2. For these female Veterans, pregnancy, breastfeeding, and/or failure to practice an effective method of birth control for the duration of participation in the study are exclusion criteria (Table 1). In the event of a positive pregnancy test, the participant will be referred to clinical care and study-related treatment will stop.

## SARS COV2 (COVID-19)

As the clinical research landscape is ever-changing during the coronavirus disease (COVID-19) pandemic, researchers must adapt in making decisions about implementing the research protocol in clinical trials. Regulations governing buprenorphine treatment were relaxed in response to the COVID pandemic to allow initiation and maintenance of buprenorphine by telemedicine without in person visits. However, due to the needs of the research to collect urine in person and the relatively low likelihood of patients presenting with active COVID infection, some in person visits were deemed necessary.

Study procedures were modified to be conducted in a manner that protects both staff and participants from COVID-19 by minimizing any risk for inadvertent transmission and by limiting exposure time. All study drug initiations, provisions of SL-BUP/NLX prescriptions, and injections of INJ-BUP are performed in person.

If potential participants are COVID-19 positive or in COVID-19 isolation, they may be given information about the study via telephone/telehealth, but may not be consented, as these procedures must occur in-person to assess and ensure informed consent. However, eligibility may be reconsidered when the patient has clinically improved.

Enrolled participants are screened per standard of care at each participating site for COVID-19 symptoms and exposure; research procedures may continue on a full face-to-face or limited face-to-face basis for those who screen negative. If an already enrolled participant is under quarantine or isolation orders, they may not be seen in person for study-related procedures. Study procedures may be rescheduled and/or conducted via telephone/telehealth. Study visit assessments and procedures that may be conducted via telehealth/telephone have been identified and include predominantly self-report measures. In order to capture information about COVID-19 and its effect on drug use, a COVID-19 questionnaire has been added to the protocol for administration at baseline and every 3 months.

## Analytic plan

The primary objective is to determine if long-acting injectable formulation of buprenorphine is superior to the sublingual formulation. There are two complementary primary endpoints: retention on study assigned MOUD and opioid abstinence. For the retention outcome, a time-to-event analysis using a two-sided log-rank test will compare the treatments. For the abstinence outcome, the mean number of opioid free UTOX screens in conjunction with self-reported abstinence at 28 distinct time points in each treatment arm will be compared by a Student t-test. Both measures must be negative for classification as abstinent. We will accrue over the follow-up period the total count of such self-report consistent opioid free urine tests. In secondary analyses UTOX samples obtained during study visits will be compared to self-reports of opioid use. All secondary analyses are exploratory and therefore not powered. All analyses of these outcome variables will be done with a single predictor, formulation (simple or one variable), or with formulation and a stock of covariates (multivariable) that predict the outcome. Predictive variable will be used for secondary analyses in which the outcomes used in the primary analyses will be assessed based upon explanatory variables that might affect treatment group outcomes between the two intervention groups.

All clinical data and study documents will be monitored by the CSP Coordinating Center (CSPCC) using an electronic data capture and clinical trial management system and follow existing regulations and standard operating procedures in handling study and participant information and used in VA Cooperative Studies.

### Sample size and power analysis

The sample size estimates primarily arise from the assumption of an overall at least 40% retention after 52 weeks. The study treatment retention times will be compared using a log-rank test, assuming that for the alternative hypothesis, the hazard ratio (injected over sublingual) is 0.74 or smaller. The two-sided hypothesis also rejects the null hypothesis if the hazard ratio is 1.36 or larger. For the comparison of abstinence, using the t-test, the alternative hypothesis posits a difference in proportions of 10% (a difference of 1.5 if mean counts are about 15). Under these assumptions, with a total size of 952 participants, this study has overall 90% power to reject both null hypotheses. Each test assumes a Type I error of 2.4% (where the interim uses 0.1%). This assumes no participants drop out (they are treatment failures), but that follow-up time ends when the participant dies or has a long-term hospitalization, is institutionalized, or incarcerated.

## Discussion

CSP #2014, VA-BRAVE, was developed soon after a new formulation of injectable buprenorphine became available for use. The study was designed as a large-scale comparative effectiveness trial designed to enroll patients with moderate to severe OUD seeking medication treatment who are enrolled in care in the VHA. The inclusion criteria are considerably broad to allow enrollment of Veterans with comorbid conditions, the co-primary outcomes of retention and abstinence were chosen as meaningful clinical measures of success, the secondary outcomes including health services and the long duration of follow-up were included to collect meaningful clinical information on the use of injectable medications in OUD. The study was designed with consideration of special populations of VHA patients with OUD, i.e., with comorbid psychiatric disorders, high rates of criminal justice involvement, infectious disease comorbidity, and/or high rates of overdose. Out of necessity, special considerations for research during the COVID-19 pandemic were also included.

Conducting this study in the VHA deserves special mention. The VHA is the largest healthcare provider in the country and currently serves approximately 81,000 Veterans with OUD annually and these numbers have continued to grow. The VA is a national system, with an electronic medical record allowing for capture of both medical and health services information, providing integrated clinical care. The robust research infrastructure of VA, and the Cooperative Studies Program specifically includes pharmacy support, data management, etc. The VA Cooperative Studies Program also has a strong track record in minority recruitment, particularly in studies of conditions prevalent in black populations [46]. Of note, this is the first CSP study to focus on OUD. Providing MOUD for OUD is a key objective for the VHA [33, 47]. This new monthly injectable formulation of buprenorphine currently represents a promising new improvement in treatment of OUD.

There are some limitations associated with this study design. In order for equipoise, all participants who are randomized to the SL-BUP/NLX arm receive a 28-day supply of medication and have an identical number of research visits as those who are randomized to the monthly INJ-BUP arm. Similarly, while INJ-BUP may have other advantages over SL-BUP/NLX such as in preventing diversion, this study will not be evaluating diversion. Finally, there is now a new injectable formulation of buprenorphine available that was not FDA approved when this study was funded [48, 49]. The question remains whether conclusions from this study will be generalizable to all injectable formulations.

## Conclusion

Positive findings in this trial could lead to widespread adoption by clinicians, that could reduce opioid use among Veterans, likely improve Veterans’ medical, psychological, and social outcomes, and undoubtedly save lives. If injectable buprenorphine is not superior to sublingual buprenorphine/naloxone, its use can be formally limited through the VA formulary for use in special circumstances. This study’s findings, positive or negative, would also be applicable to much of the non-VA opioid use disorder treatment community and would contribute substantially to the nation’s ability to respond effectively to our current opioid epidemic.

## Data Availability

Not applicable.

## Abbreviations

ASAM: American Society of Addiction Medicine
AUDIT: Alcohol Use Disorders Identification Test
CBC: Complete blood count
CIRB: Central Institutional Review Board
CIWA-Ar: Clinical Institute Withdrawal Scale
COWS: Clinical Opiate Withdrawal Scale
CSP: Cooperative Studies Program
CSPCC: Cooperative Studies Program Coordinating Center
C-SSRS: Columbia Suicide Severity Rating Scale
DO: Doctor of osteopathic medicine
EKG: Electrocardiogram
HBV: Hepatitis B
HCV: Hepatitis C
HIV: Human immunodeficiency virus
IDEAS: Informatics, Decision-Enhancement, and Analytic Sciences Center
INJ-BUP: Injectable monthly buprenorphine
MD: Doctor of medicine
MDMA: Methylene-dioxy-methamphetamine
MINI: The Mini International Neuropsychic Interview
MM: Medication management
MOUD: Medication treatments for OUD
NP: Nurse Practitioner
OEND: Overdose Education Naloxone Distribution
OUD: Opioid use disorder
PA: Physician Assistant
PARCKA: Program for Addiction Research, Clinical Care, Knowledge and Advocacy
PCL-5: Posttraumatic Stress Disorder Checklist for DSM-5
PEG3: Pain, enjoyment, general activity
PHQ-9: Patient Health Questionnaire-9
PTSD: Post-traumatic stress disorder
SL-BUP/NLX: Sublingual buprenorphine/naloxone
SURF: Service utilization review form
RCT: Randomized control trial
RN: Registered nurse
TLFB: Timeline follow-back
UTOX: Urine toxicology
NIDA: National Institute on Drug Abuse
VA: Veterans Affairs
VA-BRAVE: Buprenorphine for Treating Opioid Use Disorder in Veterans
VAS: Visual Analog Scale
VHA: Veterans Healthcare Administration
XR-NTX: Extended-release injectable formulation
